# Impulsivity in first-degree relatives at risk of psychosis and mania: a systematic review and meta-analysis

**DOI:** 10.1017/S0033291724001752

**Published:** 2024-10

**Authors:** Jess Kerr-Gaffney, Yahufu Nuerzati, Emma I. Kopra, Allan H. Young

**Affiliations:** 1 Psychology, and Neuroscience, Institute of Psychiatry, King's College London, London, UK; 2 South London and Maudsley NHS Foundation Trust, Bethlem Royal Hospital, London, UK

**Keywords:** bipolar disorder, impulsivity, mania, psychosis, schizophrenia

## Abstract

Impulsivity is elevated in psychosis and during mania in bipolar disorder. Studies in unaffected relatives may help establish whether impulsivity is a heritable, state independent endophenotype. The aim of this systematic review and meta-analysis was to examine whether impulsivity is elevated in unaffected relatives of those with bipolar disorder, schizophrenia, and schizoaffective disorder, compared to controls. Databases were systematically searched up until March 2023 for articles reporting data on a behavioral or self-report measure of impulsivity in first-degree relatives and controls. Nineteen studies were included. Behavioral (10 studies, *d* = 0.35, *p* < 0.001) and self-reported impulsivity was significantly elevated in bipolar disorder relatives compared to controls (5 studies, *d* = 0.46, *p* < 0.001), with small effect sizes. Relatives of those with schizophrenia did not show significantly elevated impulsivity compared to controls on behavioral measures (6 studies, *d* = 0.42, *p* = 0.102). There were not enough studies to conduct a meta-analysis on self-report data in schizophrenia relatives or schizoaffective disorder relatives (self-report or behavioral). Study quality was good, however there was moderate to high heterogeneity in behavioral meta-analyses. Results suggest elevated impulsivity may be an endophenotype for bipolar disorder, present in an attenuated state before and after the illness and in at-risk individuals. This trait, amongst other behavioral and psychological indices, could be used to identify those who are at risk of developing bipolar disorder. Future research should refine measurement across studies and establish which components of impulsivity are affected in those at risk of psychotic and bipolar disorders.

## Introduction

Recently, there has been a shift toward characterizing psychopathology based on neurobiological measures and observable behavior, rather than solely on traditionally defined diagnostic categories (Cuthbert & Insel, 2013). In particular, there is increasing evidence for genetic, biological, and phenomenological similarities between schizophrenia and bipolar disorder, with some proposing a continuum model to represent diagnostic variation across these disorders, with schizoaffective disorder lying in between the two (Keshavan et al., 2011). Genetic studies support substantial genetic overlap between these disorders (Lichtenstein et al., 2009).

Both schizophrenia and bipolar disorder are associated with affective and cognitive impairments, but these can differ in presentation. Schizophrenia is often characterized by emotional blunting, while bipolar disorder is characterized by depressive, mixed, and manic episodes. On the other hand, while delusional beliefs and hallucinations are core symptoms of schizophrenia, they often occur during manic episodes in bipolar disorder. Similarly, while cognitive impairments in executive functioning, attention, and memory are well-characterized in schizophrenia (Bowie & Harvey, 2006), there is increasing recognition of similar cognitive difficulties in bipolar disorder, albeit in an attenuated form (Keramatian, Torres, & Yatham, 2021). Although most pronounced during acute illness, individuals with bipolar disorder and schizophrenia demonstrate persistent, trait-like cognitive deficits in periods of remission (Bora, Yucel, & Pantelis, 2009; Nuechterlein, Ventura, Subotnik, & Bartzokis, 2014). There is evidence to suggest that cognitive impairments are predictive of later functional outcomes in both disorders (Ehrminger et al., 2021; Nuechterlein et al., 2011).

Impulsivity is a multidimensional construct, including both cognitive and behavioral components, defined as a predisposition to react toward stimuli in a rapid and unplanned manner, without regard to the consequences (Lombardo et al., 2012). Impulsivity is an important clinical feature of several psychiatric disorders, for example, it is a key feature of mania in bipolar disorder, but is also present during euthymia (Ramirez-Martin, Ramos-Martin, Mayoral-Cleries, Moreno-Kustner, & Guzman-Parra, 2020; Santana, Kerr-Gaffney, Ancane, & Young, 2022). Evidence suggests that impulsivity in bipolar disorder is associated with a more severe course of illness, higher rates of hospitalization, increased rates of relapse, increased risk of suicide, and reduced quality of life (Baldaçara et al., 2011; Jimenez et al., 2012; Oquendo et al., 2010; Victor, Johnson, & Gotlib, 2011). Due to its apparent state independence (present during euthymia as well as mania), some have suggested that impulsivity may represent an endophenotype for bipolar disorder (Bora et al., 2009).

Elevated impulsivity has also been reported in those with schizophrenia and schizoaffective disorder (Nolan, D'Angelo, & Hoptman, 2011). Impulsivity has been identified as a risk factor for violent behavior in people with psychosis (Witt, van Dorn, & Fazel, 2013), and, similar to bipolar disorder, increased risk of substance use disorder and suicide (Dervaux et al., 2001; Meltzer, 2001; Oquendo et al., 2010). However, there is some evidence to suggest different mechanisms behind elevated impulsivity in schizophrenia and bipolar disorder. Nanda et al. (2016) reported that volumes of sub-regions within the orbitofrontal cortex (OFC) were significantly inversely associated with impulsivity in both schizoaffective disorder and psychotic bipolar disorder, but not schizophrenia. The OFC may influence ‘top-down’ cognitive control over emotional reactivity and decision making, and deficits in this area may lead to failure of inhibition (Joseph, Liu, Jiang, Lynam, & Kelly, 2009; Siever, 2008). Increased impulsivity may therefore be rooted in different mechanisms in psychotic disorders with and without prominent affective components (Nanda et al., 2016).

Although elevated impulsivity has been shown to be stable across illness stages across the psychosis and bipolar spectrum, it may be that enduring subthreshold symptoms, medication, and chronic illness effects contribute to elevated impulsivity during euthymia or recovery. Therefore, studies in unaffected relatives are required to establish whether impulsivity may be a heritable, state independent endophenotype, and not a ‘scar’ of the illness. Alternatively, it may be that elevated impulsivity is shared among affected individuals but not among those at genetic risk, suggesting a phenotypic, rather than endophenotypic overlap. Thus, the aim of this systematic review and meta-analysis was to examine whether impulsivity is elevated in unaffected relatives of those with bipolar disorder, schizophrenia, and schizoaffective disorder, compared to HCs.

## Methods

### Systematic review protocol

This systematic review and meta-analysis were performed in accordance with the Preferred Reporting Items for Systematic Reviews and Meta-Analyses (PRISMA) guidelines (Page et al., 2021). The protocol was preregistered on PROSPERO (ID: CRD42023393115). There was one change to the original protocol: RStudio was used for the meta-analysis instead of RevMan.

### Information sources and search strategy

Three electronic databases (PubMed, PsycInfo, and Web of Science) were searched for papers up until 12^th^ March 2023. Searches were performed using the following terms: ‘bipolar disorder’ OR schizophrenia OR ‘schizoaffective disorder’ AND proband* OR sibling* OR mother OR father OR parent OR brother OR sister OR FDR OR ‘first degree relative’ AND impulsiv* OR ‘delay* gratification’ OR ‘inattention’ OR ‘response inhibition’. No search limits were applied. Reference lists of relevant review articles and eligible papers were also searched.

### Inclusion and exclusion criteria

Inclusion criteria for studies were: (a) case–control, cohort, or cross-sectional studies that include a group of first-degree relatives of patients with bipolar disorder, schizoaffective disorder, or schizophrenia, as well as a group of unaffected healthy controls, (b) report impulsivity data (self-report or behavioral measures) for both groups, (c) include participants that are aged ⩾18, (d) full-text in English is published in a peer-reviewed journal. Papers including participants under the age of 18 were excluded, as were case reports, reviews, or qualitative studies.

### Study selection

**Figure 1.**
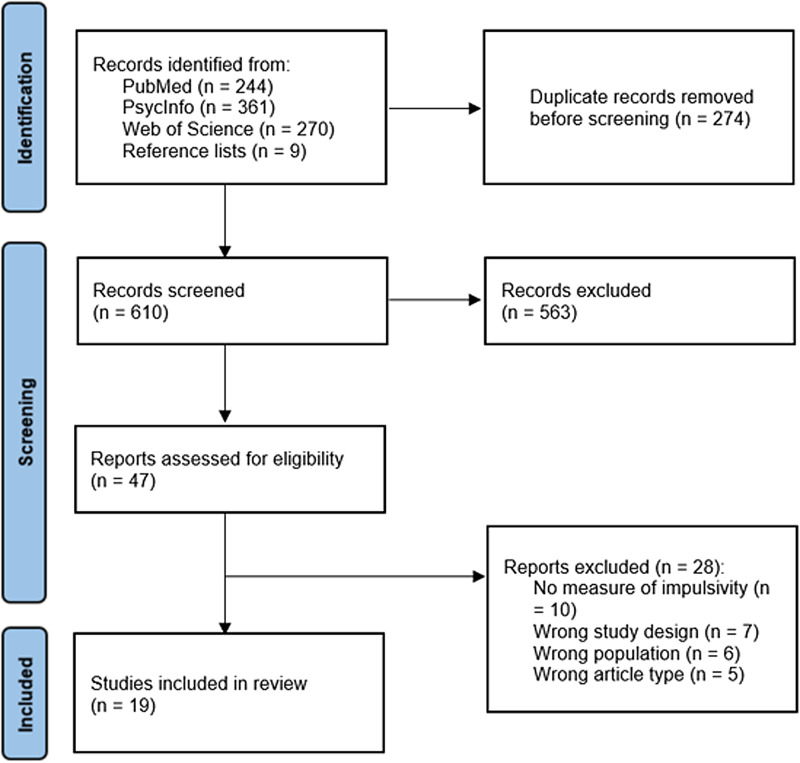
Systematic review search process.

Screening and selection of articles is displayed in Fig. 1. After removal of duplicates, titles and abstracts of 610 articles were screened by one author (YN). Where titles and abstracts appeared relevant, these were retained and full texts retrieved. 47 full-text articles were assessed for eligibility independently by two authors (YN and JKG). If an article met all eligibility criteria but did not provide data on the impulsivity measure, study authors were contacted for this information. 19 articles met all eligibility criteria and were included in the review.

### Data extraction

The following information was extracted from each article: number of participants in each group, mean age, gender, impulsivity measure and outcome variable, mean and standard deviation impulsivity score, and a summary of key findings.

### Statistical analysis

Analyses were performed using the metafor package (Viechtbauer, 2010) in Rstudio (R Core Team, 2019). Meta-analyses (for each relative subgroup *v.* HCs, split by self-report or behavioral measurement) were carried out using random-effects models. Cohen's d was used to estimate effect sizes and is reported with 95% confidence intervals (CIs). Effect sizes are interpreted using Cohen's (1988) definitions of small (0.2), medium (0.5), and large (0.8). Positive effect sizes indicate higher impulsivity in the relative group compared to the HC group. Between study heterogeneity was assessed using Cochran's *Q* test and quantified using *I*^2^, where *I*^2^ < 25% indicates low heterogeneity, 25–75% indicates moderate heterogeneity, and *I*^2^ ⩾ high heterogeneity (Higgins, 2003).

### Risk of bias

Risk of publication bias across studies was assessed via visual inspection of funnel plots, and formally using Begg and Mazumdar's (1994) rank correlation test.

Risk of bias for individual studies was assessed using the Kmet standard quality assessment criteria (Kmet, Lee, & Cook, 2004). The Kmet assesses quality of studies based on 14 criteria related study objectives, design, methodology, sample selection, reporting of the results and conclusions. Eleven of the criteria were used in this review, as three of the criteria were only applicable to randomized controlled trials (random allocation and blinding of investigators and participants to interventions). Each criterion was rated as 2 (fully met criteria), 1 (partially meeting criteria), or 0 (failing to meet criteria). A total score was then calculated, then divided by the total possible score (22) to give a summary score ranging from 0 to 1, with higher scores indicating higher quality. Risk of bias assessments were carried out independently by two authors (YN and EK), then any discrepancies resolved by a third author (JKG).

## Results

### Study characteristics

Study characteristics are shown in Table 1. Fourteen studies included a group of first-degree relatives of bipolar disorder probands, eight included a group of first-degree relatives of schizophrenia probands, and two included a group of first-degree relatives of schizoaffective disorder probands. Most relative groups were siblings only (9 studies) or a mix of siblings, parents, and offspring (7 studies), one study included offspring only, and two studies did not specify the relative composition. Seven studies used a self-report measure of impulsivity (the Barratt Impulsiveness Scale version 11 or 11A in six studies, and the Chapman Impulsive Nonconformity Scale in one study), while 13 used a behavioral measure, with the stop signal task (SST) being most common (5 studies).

**Table 1.** Characteristics of included studies

Study	Sample	Age (mean)	Sex (% female)	Impulsivity measure	Outcome measure, mean (SD)
Bauer et al. (2016)	15 BD relatives	37.5	73.3%	Affective go no-go task	Commission errors (shift): 4.60 (3.02)
	23 HC	33.5	65.2%		Commission errors (shift): 6.04 (3.81)
Bora et al. (2008)	34 BD relatives	45.7	58.8%	Continuous performance test-II	Commission errors: 11.1 (6.0)
	25 HC	47.6	64.0%		Commission errors: 8.7 (5.6)
Christodoulou et al. (2012)	17 BD relatives	38.7	76.5%	Hayling sentence completion task	Type A errors: 1.3 (2.7)
	15 SZ relatives	45.8	73.3%		Type A errors: 2.8 (2.5)
	23 HC	39.0	73.9%		Type A errors: 0.1 (0.3)
Ethridge et al. (2014)	224 SZ relatives	43.0	69.0%	Stop signal task	SSRT: 233.95 (31.72)
	194 BD-P relatives	40.0	65.0%		SSRT: 232.69 (32.94)
	44 SAD-D relatives	39.0	71.0%		SSRT: 230.24 (31.21)
	105 SAD-BP relatives	40.0	68.0%		SSRT: 233.28 (29.67)
	223 HC	38.0	58.0%		SSRT: 227.14 (28.29)
Fekih-Romdhane et al. (2022)	55 SZ relatives	27.4	54.5%	BIS-11	Total score: 62.7 (4.6)
	73 HC	28.2	59.2%		Total score: 57.9 (3.8)
Ferrier, Chowdhury, Thompson, Watson, and Young (2004)	17 BD relatives	34.2	58.8%	CANTAB sustained attention	Commission errors: 1.47 (1.97)
	17 HC	34.8	58.8%		Commission errors: 1.59 (1.97)
Finkelstein, Cannon, Gur, Gur, and Moberg (1997)	15 SZ relatives	30.5	40.0%	Continuous performance test	Commission errors: 7.8 (10.4)
	29 HC	27.3	31.0%		Commission errors: 1.9 (2.6)
Frangou, Haldane, Roddy, and Kumari (2005)	15 BD relatives	27.2	66.7%	Hayling sentence completion task	Type A errors: 6.53 (0.74)
	43 HC	42.9	44.2%		Type A errors: 4.77 (1.85)
Henna et al. (2013)	14 BD relatives	49.9	78.6%	BIS-11A	Total score: 54.6 (9.9)
	136 HC	37.1	64.7%		Total score: 53.2 (9.1)
Hidiroglu et al. (2013)	25 BD relatives	40.2	68.0%	BIS-11	Total score: 57.40 (10.10)
	30 HC	35.7	63.3%		Total score: 52.90 (7.25)
Hidiroğlu et al. (2015)	30 BD relatives	31.0	53.4%	Stop signal task	SSRT: 291.87 (56.75)
	33 HC	32.3	63.6%		SSRT: 256.32 (46.68)
Lindberg et al. (2016)	21 SZ relatives	31.5	57.1%	Stop signal task	SSRT: 187.09 (19.95)
	31 HC	30.3	48.4%		SSRT: 192.44 (24.41)
Lombardo et al. (2012)	57 BD relatives	31.4	63.2%	BIS-11	Total score: 58.90 (11.5)
	49 HC	30.4	57.1%		Total score: 52.40 (8.9)
Mathias De Almeida, Nery, Moreno, Gorenstein, and Lafer (2013)	67 BD relatives	39.0	58.0%	BIS-11	Total score: 59.94 (8.55)
	70 HC	36.8	70.0%		Total score: 57.13 (6.80)
Reilly et al. (2017)	303 SZ relatives	43.1	69.6%	Dot probe expectancy task	AY errors: 0.14 (0.16)
	187 SAD relatives	40.6	69.5%		AY errors: 0.18 (0.15)
	251 BD relatives	40.6	66.1%		AY errors: 0.14 (0.15)
	308 HC	37.7	55.5%		AY errors: 0.13 (0.15)
Thaker et al. (1993)	28 SZ relatives	44.4	57.1%	Chapman impulsive nonconformity scale	Total: 7.4 (4.7)
	22 HC	31.1	59.1%		Total: 10.0 (5.3)
Trivedi et al. (2008)	10 BD relatives	30.1	10.0%	Continuous performance test	Commission errors: 2.00 (1.49)
	10 HC	29.3	20.0%		Commission errors: 0.50 (0.53)
Wessa, Kollmann, Linke, Schonfelder, and Kanske (2015)	27 BD relatives	31.8	51.9%	BIS-11, Stop signal task	Total score: 58.04 (5.31); SSRT: 175.45 (38.90)
	29 HC	32.9	51.7%		Total score: 54.26 (6.43); SSRT: 163.80 (44.70)
Zandbelt, Van Buuren, Kahn, and Vink (2011)	24 SZ relatives	29.9	45.8%	Stop-Signal Anticipation Task	Successfully inhibited trials: 45% (4.7%)
	25 HC	32.3	37.5%		Successfully inhibited trials: 46% (5.6%)

s.d., standard deviation; BD, bipolar disorder; HC, healthy control; SZ, schizophrenia; BD-P, bipolar disorder with a history of psychosis; SAD, schizoaffective disorder; SAD-D, schizoaffective disorder, depressed type; SAD-BP, schizoaffective disorder, bipolar disorder type; SSRT, stop signal reaction time; BIS-11, Barrett Impulsiveness Scale-11; BIS-11A, Barrett Impulsiveness Scale-11A; CANTAB, Cambridge Neuropsychological Test Automated Battery.

### Synthesis of results

#### Relatives of bipolar disorder

**Figure 2.**
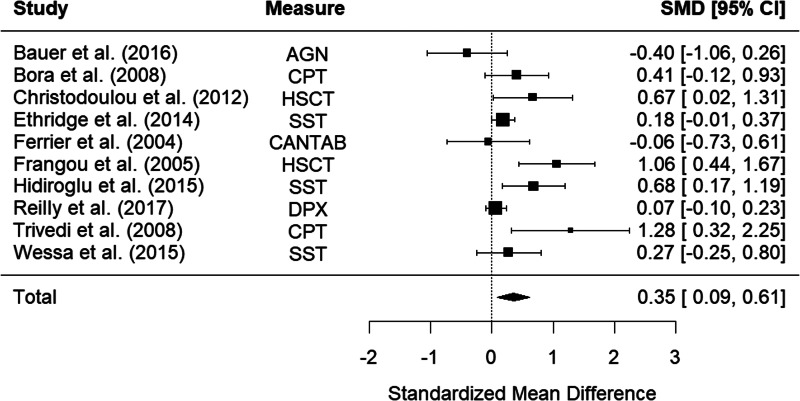
Forest plot of standardized mean differences (SMD) between bipolar disorder relatives and healthy controls (HCs) in studies assessing impulsivity with behavioral measures. Positive effect sizes indicate higher impulsivity in the relative group.

Ten studies examined impulsivity in bipolar disorder relatives using behavioral measures. The random-effects model with a total sample size of 1344 participants (610 bipolar disorder relatives and 734 HCs) showed that relatives of those with bipolar disorder were significantly more impulsive than HCs, with a small effect size, *d* = 0.35, 95% CI 0.09–0.61, *z* = 2.67, *p* < 0.001 (Fig. 2). There was moderate heterogeneity between studies, *Q*(9) = 24.60, *p* = 0.003, *I*^2^ = 72.11%.

**Figure 3.**
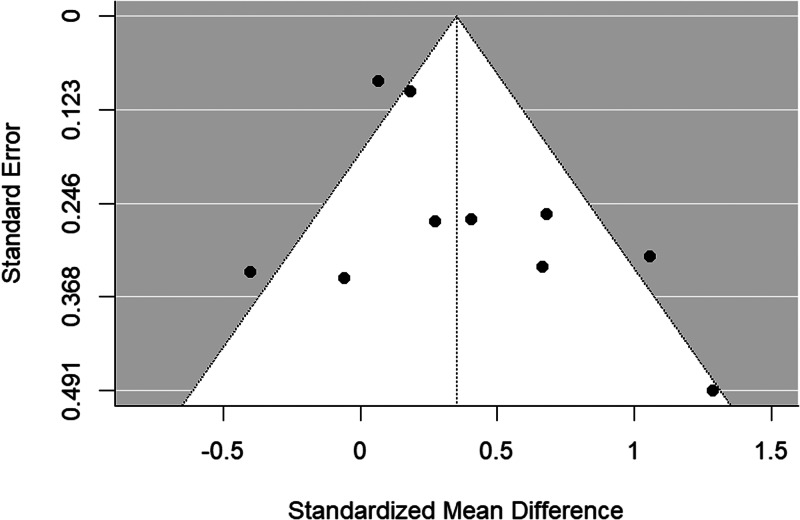
Funnel plot of bipolar disorder relatives *v.* HC behavioral studies included in the meta-analysis.

The funnel plot is shown in Fig. 3. There was no evidence of significant publication bias (Begg's test *p* = 0.484).

**Figure 4.**
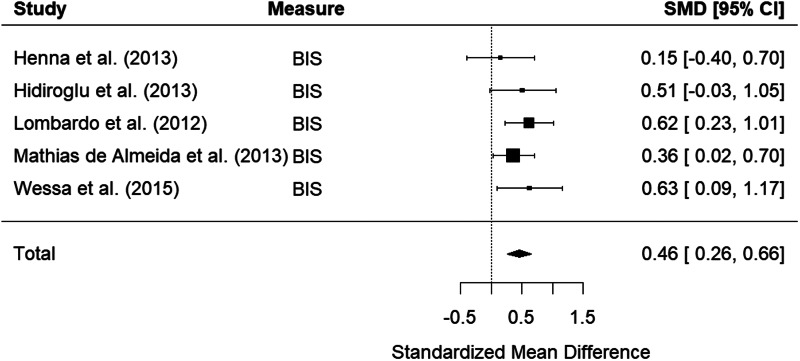
Forest plot of standardized mean differences (SMD) between bipolar disorder relatives and healthy controls (HCs) in studies assessing impulsivity with self-report measures. Positive effect sizes indicate higher impulsivity in the relative group.

Five studies examined self-report measures of impulsivity in bipolar relatives. The random-effects model with a total sample size of 504 participants (190 bipolar relatives and 314 healthy controls) showed that relatives of those with bipolar disorder were significantly more impulsive than HCs, with a small effect size, *d* = 0.46, 95% CI 0.26–0.66, *z* = 4.54, *p* < 0.001 (Fig. 4). There was low heterogeneity between studies, *Q*(4) = 2.60, *p* = 0.626, *I*^2^ = 0.00%.

**Figure 5.**
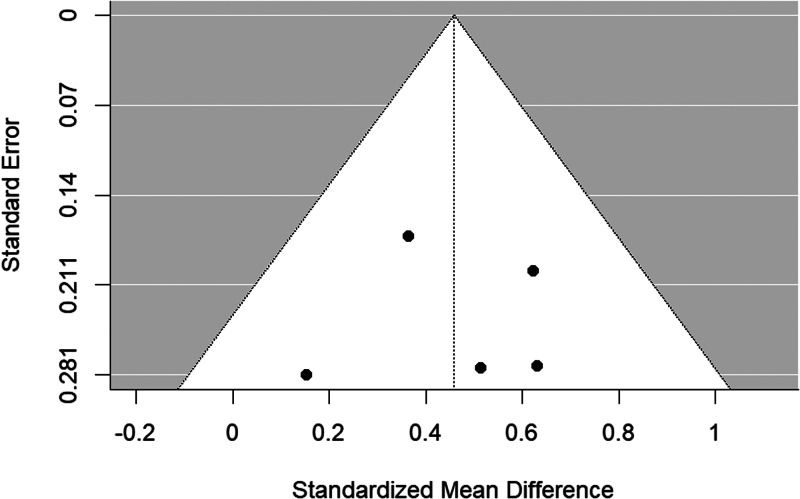
Funnel plot of bipolar disorder relatives *v.* HC self-report studies included in the meta-analysis.

The funnel plot is shown in Fig. 5. There was no evidence of significant publication bias (Begg's test *p* = 0.483).

#### Relatives of schizophrenia

**Figure 6.**
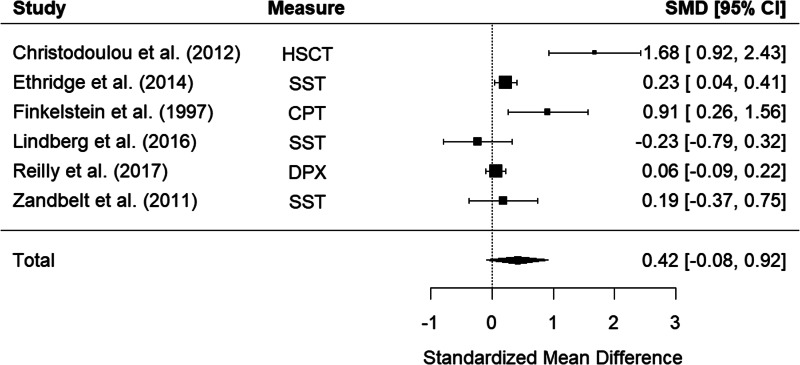
Forest plot of standardized mean differences (SMD) between schizophrenia relatives and healthy controls (HCs) in studies assessing impulsivity with behavioral measures. Positive effect sizes indicate higher impulsivity in the relative group.

Six studies examined impulsivity in schizophrenia relatives using behavioral measures. The random-effects model with a total sample size of 1241 participants (603 schizophrenia relatives and 638 HCs) showed that relatives of those with schizophrenia did not show significantly elevated impulsivity compared to HCs, *d* = 0.42, 95% CI −0.08 to 0.92, *z* = 1.64, *p* = 0.102 (Fig. 6). There was high heterogeneity between studies, *Q*(5) = 24.43, *p* < 0.001, *I*^2^ = 92.33%.

**Figure 7.**
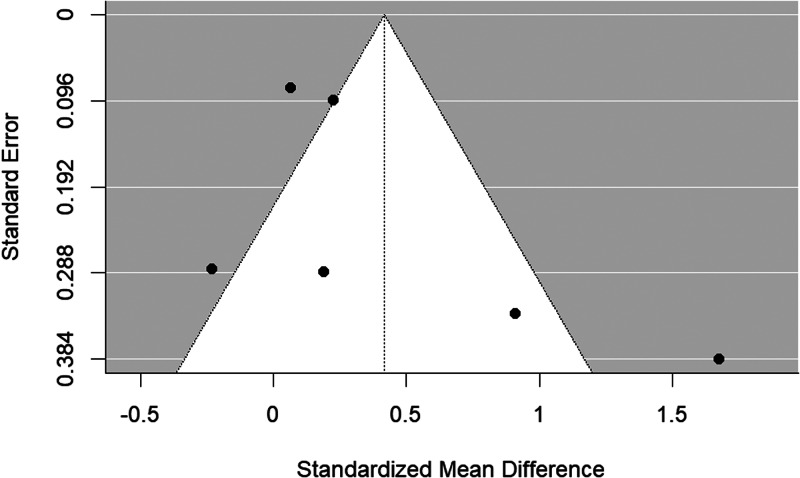
Funnel plot of schizophrenia relatives *v.* HC behavioral studies included in the meta-analysis.

The funnel plot is shown in Fig. 7. There was some evidence of publication bias (Begg's test *p* = 0.056).

Two studies examined self-reported impulsivity in relatives of those with schizophrenia, therefore a meta-analysis could not be conducted. Thaker, Moran, Adami, and Cassady (1993) administered the Chapman Psychosis Proneness Scales, a group of scales used to identify schizotypal phenotypes in non-psychotic individuals, to relatives of those with schizophrenia, HCs, and relative and non-relative groups with personality disorders. The Impulsive Nonconformity Scale measures unorthodoxy, impulsivity, and insensitivity to the feelings of others. Though scores were slightly higher in the HC group (mean = 10.0, s.d. = 5.3) than the relative group (mean = 7.4, s.d. = 4.7), this difference was not statistically significant. Fekih-Romdhane, Maktouf, and Cheour (2022) administered the Barratt Impulsiveness Scale to patients with first-episode schizophrenia, siblings of those with schizophrenia, and HCs. Siblings showed significantly higher scores on the non-planning subscale compared to HCs but did not differ on attentional or motor subscales.

#### Relatives of schizoaffective disorder

Only two studies included relatives of people with schizoaffective disorder, both using behavioral measures, therefore a meta-analysis could not be performed. Ethridge et al. (2014) examined performance on the SST in relatives of people with schizophrenia, bipolar disorder with a history of psychosis, schizoaffective disorder depressed-type, schizoaffective bipolar-type, and HCs, finding no differences in performance in any relative group compared to HCs when controlling for generalized cognitive impairment. Similarly, Reilly et al. (2017) did not find differences in false alarm rates on the dot probe expectancy task in relatives of those with schizophrenia, psychotic bipolar disorder, or schizoaffective disorder, compared to HCs.

### Risk of bias

The full risk of bias rating for all included studies is shown in the online Supplementary material. Generally, study quality was high; summary scores ranged from 0.77 (Henna et al., 2013) to 1.00 (Fekih-Romdhane et al., 2022; Lombardo et al., 2012; Reilly et al., 2017). Objectives, study designs, and outcome measures were appropriate and sufficiently described (likely due in part to our inclusion criteria specifying measures, outcome reporting, and study design), and results and conclusions were supported and reported in detail. Study quality was more variable with regard to recruitment methods, sample size, and control of confounding variables.

## Discussion

The aim of this systematic review and meta-analysis was to examine whether impulsivity is elevated in unaffected first-degree relatives of those with bipolar disorder, schizophrenia, and schizoaffective disorder, representing a putative trait-marker in at-risk populations. Our results suggested that impulsivity is significantly elevated in relatives of those with bipolar disorder compared to HCs, with a small effect size. This was true for both self-reported impulsivity and behavioral measures. Although a similar effect size was found in studies assessing impulsivity with behavioral measures in relatives of those with schizophrenia compared to HCs, this effect was not significant. There was not enough evidence to synthesize findings from studies assessing self-reported impulsivity in schizophrenia relatives or in relatives of patients with schizoaffective disorder (behavioral or self-report); individual studies did not find strong evidence of elevated impulsivity in either population.

Previous meta-analyses have reported elevated impulsivity during euthymia in those with bipolar disorder (with medium to large effect sizes), suggesting impulsivity may be a trait feature of the disorder, present before and after illness onset (Santana et al., 2022). Our results extend these findings, showing elevated impulsivity, albeit, in an attenuated form, in at-risk populations. Together, these results indicate that impulsivity may be a heritable, state independent endophenotype for bipolar disorder. Indeed, impulsivity has a genetic basis, and similar to other complex traits, a large number of genetic variants seem to influence it's expression, each with small individual effects (Bezdjian, Baker, & Tuvblad, 2011; Khadka et al., 2014). In particular, some studies have found that genetic dopaminergic variation is associated with differences in impulsivity in the general population (Colzato, van den Wildenberg, Van der Does, & Hommel, 2010; Congdon, Lesch, & Canli, 2008). Others have reported associations between BDNF and serotonin transporter (5-HTT) protein polymorphisms and impulsivity in bipolar disorder and HCs (Boscutti et al., 2022), but no interactions between diagnosis and polymorphism status, suggesting comparable effects of these genes among those with and without bipolar disorder.

Although we did not find a significant difference in impulsivity assessed via behavioral measures between relatives of patients with schizophrenia and controls, we cannot rule out the possibility that impulsivity is elevated in this population, given the relatively small number of studies included. The effect size (*d* = 0.42) was very similar to that found in bipolar disorder relatives (*d* = 0.35), but there was greater variability in results across studies. More studies in relatives of those with schizophrenia and schizoaffective disorder are required to establish whether impulsivity may represent an endophenotype for these disorders, as in bipolar disorder. Importantly, consistency of measurement across studies is required, as currently studies use a wide array of measures to assess different aspects of impulsivity, leading to variability in results. Unfortunately, it was not possible to perform a moderator analysis with each different measure as a factor level, due to the small number of included studies and large number of measures used. Establishing which measures are related to functional impairment, or detrimental behaviors (e.g. substance use, self-harm) will also be an important avenue for future research.

Nonetheless, it may be that elevated impulsivity in those with schizophrenia is, at least partially, a consequence of the acute illness, or that different mechanisms underlie impulsivity in bipolar disorder and schizophrenia. Indeed, impulsivity appears to be less pronounced in younger patients with early onset, first episode schizophrenia spectrum disorders (Jepsen et al., 2018). Some have suggested that impulsive responding in behavioral tasks (such as response inhibition) seen in those with schizophrenia is due to generalized cognitive deficits, rather than impulsivity *per se* (Christodoulou, Messinis, Papathanasopoulos, & Frangou, 2012). Response inhibition tends to be particularly affected in bipolar disorder, more so than schizophrenia or schizoaffective disorder, and appears to represent a specific cognitive deficit rather than a generalized one, as in the psychotic disorders (Ethridge et al., 2014). Furthermore, in a large sample of patients with bipolar disorder, schizophrenia, and schizoaffective disorder and their relatives, Ethridge et al. (2014) reported that SST performance was associated with social adjustment and self-reported impulsivity in bipolar disorder only, suggesting that reduced inhibitory control may contribute to behavioral impulsivity and social difficulties in this population. In sum, although first-degree relatives and patients across diagnoses may show elevated impulsivity (with strongest evidence in bipolar disorder and their relatives), the mechanisms underlying these similarities are likely to differ across diagnoses.

To our knowledge, this is the first review to synthesize studies assessing impulsivity in relatives of those with bipolar disorder, schizophrenia, and schizoaffective disorder. Studies in first-degree relatives are useful in distinguishing trait *v.* state features, as relatives are generally medication-free, and have not experienced repeated episodes of the illness in question, which may have lasting effects on behavior and cognition. However, several limitations should be noted. Firstly, the small number of studies in schizophrenia and schizoaffective disorder prevented us from making firm conclusions regarding impulsivity as an endophenotype in these disorders. As previously mentioned, studies included a wide array of measures, many of which assessed different aspects of impulsivity (e.g. response inhibition, inattention). The small number of studies included prevented separate sub-analyses of these aspects, as has been done in previous reviews in probands (Ramirez-Martin et al., 2020; Santana et al., 2022). Finally, included studies sometimes did not control for confounding variables or did not match relative and control groups on key demographic variables, which may have introduced bias to our results.

Our findings suggest elevated impulsivity may be a putative endophenotype for bipolar disorder, present in an attenuated state before and after the illness and in at-risk individuals. This trait, amongst other behavioral and psychological indices, could be used to identify those who are at risk of developing bipolar disorder. Indeed, higher impulsivity in those at risk of bipolar disorder has been shown to predict bipolar disorder diagnosis 13 years later (Kwapil et al., 2000). In turn, early interventions could be targeted to high-risk groups, for example, cognitive interventions that target aspects of impulsivity may be beneficial in improving outcomes (Tsapekos, Strawbridge, Cella, Young, & Wykes, 2023). There was not enough evidence to conclude whether impulsivity is also elevated in relatives of those with schizophrenia and schizoaffective disorder. Future research should refine measurement across studies and establish which components of impulsivity are affected in those at risk of psychotic and bipolar disorders.

## Supporting information

Kerr-Gaffney et al. supplementary material Kerr-Gaffney et al. supplementary material

## Supplementary material

The supplementary material for this article can be found at https://doi.org/10.1017/S0033291724001752
