# Supplementary material for: Impulsivity in first-degree relatives at risk of psychosis and mania: a systematic review and meta-analysis
Source: Psychol Med. 2024 Oct 14;54(13):3572–80. doi: 10.1017/S0033291724001752 (PMC11536112; doi:10.1017/S0033291724001752)
Supplement: Kerr-Gaffney et al. supplementary material [file S0033291724001752sup001.docx]

Table S1. Quality assessment for included studies.

| **Study** | **1** | **2** | **3** | **4** | **5** | **6** | **7** | **8** | **9** | **10** | **11** | **Summary score** |
| --- | --- | --- | --- | --- | --- | --- | --- | --- | --- | --- | --- | --- |
| Bauer et al. (2016) | 2 | 2 | 1 | 2 | 2 | 1 | 2 | 2 | 2 | 2 | 2 | 0.91 |
| Bora et al. (2008) | 2 | 2 | 2 | 2 | 1 | 1 | 2 | 2 | 2 | 2 | 2 | 0.91 |
| Christodoulou et al. (2012) | 2 | 2 | 2 | 2 | 2 | 1 | 2 | 2 | 2 | 2 | 2 | 0.95 |
| Ethridge et al. (2014) | 2 | 2 | 1 | 1 | 2 | 2 | 2 | 2 | 1 | 2 | 2 | 0.86 |
| Fekih-Romdhane et al. (2022) | 2 | 2 | 2 | 2 | 2 | 2 | 2 | 2 | 2 | 2 | 2 | 1.00 |
| Ferrier et al. (2004) | 2 | 2 | 0 | 2 | 2 | 1 | 2 | 2 | 2 | 2 | 2 | 0.86 |
| Finkelstein et al. (1997) | 2 | 2 | 2 | 2 | 2 | 1 | 1 | 2 | 1 | 2 | 2 | 0.86 |
| Frangou et al. (2005) | 2 | 2 | 1 | 2 | 2 | 1 | 2 | 2 | 2 | 2 | 2 | 0.91 |
| Henna et al. (2013) | 1 | 2 | 2 | 2 | 2 | 1 | 1 | 2 | 0 | 2 | 2 | 0.77 |
| Hidiroglu et al. (2013) | 2 | 2 | 2 | 2 | 2 | 1 | 2 | 2 | 2 | 2 | 2 | 0.95 |
| Hidiroglu et al. (2015) | 2 | 2 | 1 | 2 | 2 | 1 | 2 | 2 | 2 | 2 | 2 | 0.91 |
| Lindberg et al. (2016) | 2 | 2 | 1 | 2 | 2 | 1 | 2 | 2 | 2 | 2 | 2 | 0.91 |
| Lombardo et al. (2012) | 2 | 2 | 2 | 2 | 2 | 2 | 2 | 2 | 2 | 2 | 2 | 1.00 |
| Mathias de Almeida et al. (2013) | 2 | 2 | 2 | 2 | 2 | 2 | 2 | 2 | 1 | 2 | 2 | 0.95 |
| Reilly et al. (2017) | 2 | 2 | 2 | 2 | 2 | 2 | 2 | 2 | 2 | 2 | 2 | 1.00 |
| Thaker et al. (1992) | 2 | 2 | 2 | 1 | 1 | 1 | 2 | 2 | 1 | 2 | 2 | 0.82 |
| Trivedi et al. (2008) | 2 | 2 | 2 | 2 | 2 | 1 | 2 | 2 | 2 | 2 | 2 | 0.95 |
| Wessa et al. (2015) | 2 | 2 | 2 | 1 | 2 | 1 | 1 | 2 | 1 | 2 | 2 | 0.82 |
| Zandbelt et al. (2011) | 2 | 2 | 2 | 2 | 2 | 1 | 2 | 2 | 2 | 2 | 2 | 0.95 |

1 = Question/objective sufficiently described; 2 = Study design evident and appropriate; 3 = Method of subject/comparison group selection or source of information/input variables described and appropriate; 4 = Subject and comparison group characteristics sufficiently described; 5 = Outcome measure(s) well defined and robust to measurement/misclassification bias, means of assessment reported; 6 = Sample size appropriate; 7 = Analytic methods described/justified and appropriate; 8 = Some estimate of variance is reported for the main results; 9 = Controlled for confounding; 10 = Results reported in sufficient detail; 11 = Conclusions supported by the results.
